# Mapping the Tumor Microenvironment in TNBC and Deep Exploration for M1 Macrophages-Associated Prognostic Genes

**DOI:** 10.3389/fimmu.2022.923481

**Published:** 2022-06-30

**Authors:** Baojin Xu, Hefen Sun, Xiaoqing Song, Qiqi Liu, Wei Jin

**Affiliations:** ^1^ Key Laboratory of Breast Cancer in Shanghai, Fudan University Shanghai Cancer Center, Shanghai, China; ^2^ Department of Oncology, Shanghai Medical College, Fudan University, Shanghai, China; ^3^ Department of Breast Surgery, Liaoning Cancer Hospital and Institute, Cancer Hospital of China Medical University, Shenyang, China

**Keywords:** triple negative breast cancer, tumor immune microenvironment, macrophages, IFI35, ScRNA-seq analysis, bulk-RNA sequencing

## Abstract

Triple negative breast cancer (TNBC) remains the worst molecular subtype due to high heterogeneity and lack of effective therapeutic targets. Here we investigated the tumor and immune microenvironment heterogeneity of TNBC using scRNA-seq and bulk RNA-seq data from public databases and our cohort. Macrophage subpopulations accounted for a high proportion of tumor immune microenvironment (TIME), and M1 macrophages were associated with better clinical outcomes. Furthermore, three maker genes including IFI35, PSMB9, and SAMD9L showed a close connection with M1 macrophages. Specifically, IFI35 was positively associated with macrophage activation, chemotaxis, and migration. Also, patients with high IFI35 expression had a better prognosis. *In vitro* studies subsequently demonstrated that IFI35 was upregulated during the M1 subtype differentiation of macrophages. In summary, our data suggested that IFI35 maybe a promising novel target that helps to reshape macrophage polarization towards the M1 subtype for anti-tumor effects.

**Significance:** This study defines IFI35 as a potential therapeutic target to remodel the polarization of macrophages towards the M1 subtype in patients with TNBC.

## Introduction

Triple negative breast cancer (TNBC) accounts for 10-15% of all breast cancer cases, and lacks effective therapeutic targets due to the paucity of ER, PR, and Her2 expression (1). Recently, advances in immunotherapy such as anti-PD1, anti-PDL1, and anti-CTLA4 have dramatically ameliorated outcomes in patients with a series of solid tumors, which also brings new hope for TNBC. Currently, PD-1/PD-L1 inhibitor therapy has been approved by the U.S. Food and Drug Administration (FDA) for treating TNBC, but few patients received durable clinical responses as a result of the limitation of immunosuppressive tumor microenvironment (TME) (2). With an in-depth understanding of the tumor immune microenvironment (TIME), the most abundant macrophage subpopulation in TIME ignited our interest.

Generally speaking, macrophages can be polarized into classically activated macrophages and alternatively activated macrophages, termed M1 and M2 subsets, respectively (3, 4). On the one hand, M1 macrophages, stimulated by cytokines such as interferon gamma (IFN-γ) and lipopolysaccharide (LPS), act as phagocytic cells and antigen-presenting cells and release tumor necrosis factor-α (TNF-α), interleukin-1α (IL-1α), IL-1β, and IFN-γ, which further exert tumoricidal activity. Phenotypically, they express costimulatory molecules including CD80, CD86, and MHC II. On the other hand, in response to other cytokines such as interleukin-4 (IL-4) and IL-13, macrophages are polarized into M2 subtype that produces anti-inflammatory factors such as arginase 1, IL-10, and TGF-β, participating in angiogenesis and forming immunosuppressive microenvironment to lead to tumor growth, progression and metastasis. M2 macrophages are phenotypically characterized by CD206 and CD163 expression. At present, mounting evidence has indicated that macrophage infiltration in most tumors is dominated by the M2 subtype and is associated with tumor progression and poor patient prognosis (5–7). In TNBC, peripheral blood monocytes recruited into tumor microenvironment, in response to specific stimuli, undergo M2 activation that promotes tumor progression *via* several mechanisms including the secretion of inhibitory cytokines, the reduction of cytotoxic T lymphocytes, and the promotion of regulatory T cells (8, 9). Application of specific gene knockout technology and antibody against M2 macrophages infiltration improve the efficacy of immunotherapy (10, 11). In fact, tumor-associated macrophages (TAMs) in the TIME cannot be simply referred to as the M1/M2 dichotomy, they actually exist in the form of a mixture and change dynamically in response to different environment milieu, consequently enabling them potential targets for cancer immunotherapy.

Interferon induced protein 35 (IFI35) was first identified in HeLa cells treated with IFN-γ. It exists in the extracellular region, cytoplasm and nucleus, but does not co-localized with any organelle (12, 13). To date, a series of studies demonstrated that IFI35 can bind to Nmi or BTas after viral infection, thereby activating type I interferon antiviral response (14). While others also revealed that it negatively regulate RIG-I-mediated antiviral signaling through the ubiquitination pathway (15, 16). In addition, extracellular IFI35 and Nmi, as two damage-associated molecular patterns (DAMPs), activated NF-κB pathway *via* Toll-like receptor 4 (TLR4) from macrophages, leading to the exacerbation of inflammation-related diseases such as lupus nephritis, sepsis and multiple sclerosis (17–19). Yu et al. also identify IFI35 as a marker associated with SARS-CoV-2 or influenza virus-induced syndromes. IFI35 knockout mice and IFI35 neutralizing antibodies reduce inflammation-related lung injury (20). However, there are still very few studies on IFI35 in tumor biology, among which mainly focused on enhanced radiosensitivity of lung adenocarcinoma and colorectal cancer (21, 22). Thus, exploration for the relationship between IFI35 and macrophages polarization is an attractive direction for cancer immunotherapy.

Herein, we integrated single-cell RNA sequencing (scRNA-seq) and bulk-RNA sequencing data of TNBC to analyze the heterogeneity of the tumor and TIME. The results indicated that TAMs were highly enriched in the TIME, which reflected the potential value for remodeling TAMs. To further indentify the key genes associated with macrophages polarization, a weighted gene co-expression network analysis (WGCNA) was constructed, and correlation and survival analyzed were performed. As a result, three marker genes involved in macrophage polarization toward M1 phenotype, including IFI35, were uncovered. Ultimately, we analyzed the connection between IFI35 and M1 macrophages from two levels of cell lines and tissue samples.

## Materials and Methods

### Study Cohort

Our study included single-cell and bulk-RNA sequencing multi-omics data, in which a total of 360 TNBC patients from Fudan University Shanghai Cancer Center (FUSCC) and 158 TNBC patients from TCGA cohort were analyzed, with more detailed information previously described (23). The scRNA-seq data about 5 TNBC patients was downloaded from European Genome-phenome Archive with the study ID EGAS00001005061 (24).

### Single-Cell RNA-Seq Analysis

In the process of data processing, the ‘Seurat’ package was used and UMAP method was applied for non-linear dimensional reduction (25). Then, the combination of the ‘singleR” package and canonical marker annotated cell clusters (26). The copy number variations from malignant epithelial cells were inferred by the ‘inferCNV’ package. The ‘Monocle 3 alpha’ package was applied to infer pseudotime cell trajectory of immune cells (27).

### Bulk-RNA Sequencing Analysis

In the bulk-RNA sequencing data from FUSCC and TCGA TNBC cohorts, immune cell infiltration was estimated by the ‘CIBERSORT’ package (28). The mutational landscape between the low and high M1 macrophages infiltration group was visualized by the ‘maftools’ package (29). Weighted gene co-expression network analysis (WGCNA) was constructed, in which a power of 5 was set as soft-threshold parameter (30). The module with the highest score for M1 infiltration was identified, in which top 30 genes with high connectivity were shown.

### Correlation and Survival Analysis

Pearson’s coefficient analysis was performed to explore correlation, and the ‘Survival’ package was applied to complete survival analysis. A value of *p <*0.05 was considered to indicate a statistically significant difference.

### GSEA Enrichment Analysis

Three classical macrophage-associated gene sets were downloaded from the Molecular Signatures Database (MSigDB) (http://www.gsea-msigdb.org/gsea/msigdb/index.jsp), including GOBP_Macropahge_Activation, GOBP_Macrophage_Chemotaxis, and GOBP_Macrophage_Migration. The enrichment analysis was performed by GSEA software (version 4.1.0).

### Cell Culture

Human monocyte cell line THP-1 was purchased from Chinese Academy of Science Cell Bank (Shanghai, China; catalog no. TCHu 57). THP-1 was cultured in complete RPMI 1640 containing 10% FBS (Gibco), and 1% PenStrep and induced into M0 macrophages in the presence of 100 ng/mL PMA (Peprotech; catalog no. 1652981) for 8 hours. To obtain M1 macrophages, M0 macrophages were then treated with 100ng/ml LPS (Sigma-Aldrich; catalog no. L2630) and 20ng/ml IFN-γ (Peprotech; catalog no. 300-02). While, M2 macrophages were induced by the combination of 20ng/ml IL-4 (Peprotech; catalog no. 200-04) and 20ng/ml IL-13 (Peprotech; catalog no. 200-13). All cells were found to be negative for *Mycoplasma* upon repeated testing every month using MycoBlue Mycoplasma Detector (Vazyme; catalog no. D101) and were maintained in 37°C with a humidified atmosphere of 5% CO_2_ in air.

### RT-PCR

Total RNA was extracted by using Trizol reagent (Invitrogen; catalog no. 15596026). cDNA were synthesized from 500ng of total RNA using PrimeScript™ RT reagent Kit (Takara; catalog no. RR037A) and RT-qPCR was performed by ChamQ Universal SYBR qPCR Master Mix (Vazyme; catalog no. Q711) using QuantStudio™ 6 Flex Real-Time PCR System (Thermofisher Scientific; catalog no. 4485691) according to the manufacturer’s protocols. Gene expression was normalized relative to GAPDH. Data were analyzed by applying the 2−ΔΔC_T_ calculation method. For a detailed list of RT-qPCR primer sequences, see **Supplementary Table S1**.

### Immunoblotting

Cells were washed with PBS and lysed using RIPA cell lysis and extraction buffer (Thermofisher Scientific; catalog no. 89901) supplemented with Halt™ Protease and Phosphatase Inhibitor Cocktail (Thermofisher Scientific; catalog no. 78440). Total protein concentration was determined using Pierce™ BCA Protein Assay Kit (Thermofisher Scientific; catalog no. 23225). Proteins were separated by gradient SDS–PAGE and transferred to PVDF membranes. Blots were blocked in PBS containing 5% milk powder and 0.1% Tween, and then incubated overnight at 4°C with primary antibodies; anti-IFI35 (1:1000, Abcam; catalog no. ab233415) and anti-β-Actin (1:5000, Proteintech; catalog no. HRP-60008). After washing, appropriate HRP-conjugated secondary antibody (SAB; catalog no. L3012) was incubated for 1 hours at room temperature. Chemoluminescence was detected by Pierce ECL Western Blotting Substrate (Thermofisher Scientific; catalog no. 32209) and captured on ChemiDoc™ XRS+ System (Bio-Rad; catalog no. 1708265).

### Immunofluorescence

Cells were washed with PBS and fixed using 4% paraformaldehyde. Subsequently, cells were permeabilized using 0.5% Triton X-100 for 5 min, and blocked by protein block (Abcam; catalog no. ab64226). Then, cells were incubated overnight at 4°C with primary antibody anti-IFI35 (1:100, Abcam; catalog no. ab233415) followed by appropriate biotinylated secondary antibody (1:500, Abcam; catalog no. ab150077). Finally, cells were mounted with mounting medium with DAPI (Beyotime Biotechnology; catalog no. P0131) and images were captured on inverted microscope (Leica; catalog no. DMI6000B).

### Double-Labeling Immunohistochemistry

The paraffin-embedded TNBC specimens were sectioned in 4μm thickness. After removal of paraffin with xylenes and a graded series of alcohols, tissue sections were subjected to antigen retrieval by EDTA (Beyotime Biotechnology; catalog no. P0085) in a high temperature and pressure environment for 5 min, and then were blocked by peroxidase and alkaline phosphatase endogenous blocking solution (Vector; catalog no. SP-6000) for 15 min and protein block (Abcam; catalog no. ab64226) for 1 h, respectively. After washing with PBS buffer, the sections were incubated with primary antibody mix containing mouse anti-CD86 (1:50, Abcam; catalog no. ab220188) and rabbit anti-IFI35 (1:100, Abcam; catalog no. ab233415) overnight at 4°C. The following day, the sections were incubated for 30 min in HRP-conjugated goat-anti-mouse secondary antibody (1:500, Jackson ImmunoResearch; catalog no. 115-035-003), followed by a 1-min incubation period with peroxidase substrate (Vector; catalog no. SK-4105). After washing in in distilled water, they were then incubated with AP-conjugated horse-anti-rabbit secondary antibody (Cell Signaling Technology; catalog no. 18653) for 30 min, followed by a 20-min incubation period with alkaline phosphatase substrate (Vector; catalog no. SK-5105). Between each step the specimens were rinsed three times with TBS buffer. Nuclei were counterstained with hematoxylin. Finally, the sections were coverslipped using aqueous mounting medium (Abcam; catalog no. ab64230) and images were captured on microscope (Olympus; catalog no. BX43). All tissue samples included were approval by the Ethics Committee of Fudan University Shanghai Cancer Center (050432-4-1805C).

### Statistical Analysis

All data were analyzed by GraphPad Prism Version 9 software. Normal distribution of all data were first tested. Comparisons between two groups were made by using an unpaired two-tailed Student’s t-test. For scRNA-seq and bulk-RNA data, all statistical analysis were performed with RStudio (version 1.4.1106). All *p*-values are two-sided, and statistical significance was evaluated at the 0.05 level.

## Results

### Intra-Tumor and Inter-Tumor Heterogeneity of TNBC

**Figure 1:**
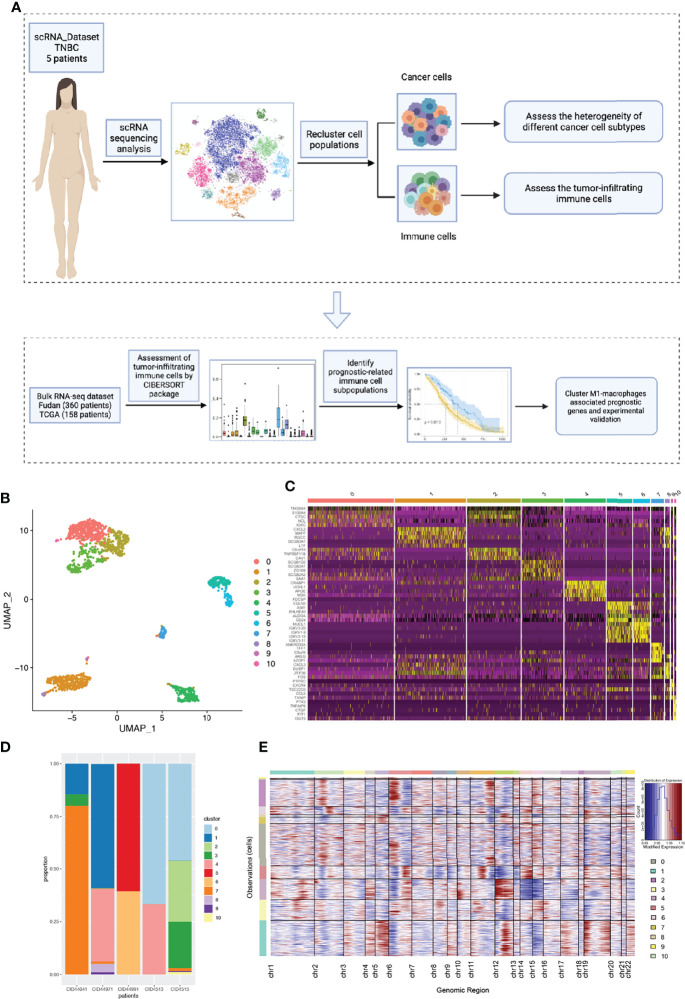
The intra-tumor and inter-tumor heterogeneity of TNBC from scRNA-seq analysis. **(A)** A brief flow chart of the analysis of the entire article is displayed. **(B)** The malignant epithelial cells were extracted and re-clustered into 11 clusters based on UMAP method. **(C)** The top5 marker genes of 11 malignant cell clusters were shown in the form of a heatmap. **(D)** All the cell clusters were detected in each patients, with varying proportions of cell types between cases. **(E)** We calculated the copy number variants in every cell clusters to estimate mutational differences within tumors.

As shown in the flowchart (**Figure 1A**), we first explored the intra- and inter-tumor heterogeneity of TNBC based on scRNA-seq analysis. Using the ‘SingleR’ package and canonical marker, we successfully annotated 12 cell clusters, including multiple immune cell clusters, epithelial cells, stromal cells, endothelial cells, and tissue stem cells (**Supplementary Figure S1**).

To further explore tumor heterogeneity, epithelial cells were divided into a total of 11 malignant cell subtypes (**Figure 1B**). Interestingly, we found that tumor cells had extensive and obvious heterogeneity, even within the same tumor type. When elucidating the characteristics of different subtypes, top five marker genes were shown in **Figure 1C**. In order to more effectively prove the existence of heterogeneity within and between tumors, the composition ratio of different cell subgroups in each patient’s tumor was depicted (**Figure 1D**). It is not difficult to find that cluster 7 accounted for more than 75% and constituted the main tumor population in patient CID44041, while this subtype was rarely found in other patients’ tumors. In addition, patient CID44971 predominantly possessed cluster 1, patient CID44991 cluster 5, and patients CID4513 and CID4515 cluster 0. Thus, varying proportions of cell subpopulations between cases were demonstrated, indicating the existence of intra-tumor and inter-tumor heterogeneity. As we all know, copy number variations (CNVs) in tumor cells are widespread. Therefore, we further explored the genomic copy number alterations in various tumor cell subtypes using inferCNV (**Figure 1E**). Cluster 4 showed high CNVs on chromosome 12, whereas cluster 2 had higher CNVs on chromosome 6. It can be seen that the heterogeneity within the tumor is not only reflected in the constituent components, but also in the form of genetic mutations.

### Complexity and Heterogeneity of the Tumor Immune Microenvironment

**Figure 2:**
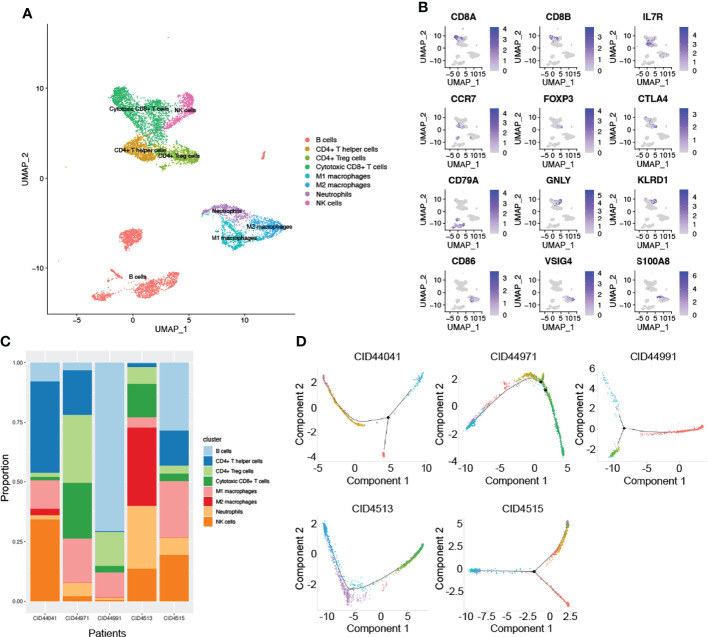
The complex composition of the tumor immune microenvironment. **(A)** Combining singleR and canonical marker, we successfully annotated 8 immune cell clusters, including B cells, CD4^+^ T helper cells, CD4^+^ Treg cells, cytotoxic CD8^+^ T cells, NK cells, neutrophils, M1 and M2 macrophages. **(B)** Representative marker genes were shown using non-linear dimensional reduction (B cells: CD79A, CD4+ T helper cells: IL7R and CCR7, NK cells: GNLY and KLRD1, M1 macrophages: CD86, neutrophils: S100A8, CD4+ Treg cells: FOXP3 and CTLA4, and M2 macrophages: CD163 and VSIG4). **(C)** All annotated immune cell clusters were estimated in each patients, with varying proportions of cell types between cases. **(D)** Pseudotime analysis was used to construct the trajectory of immune cells differentiation.

In addition to research on tumor heterogeneity, TIME has gradually become an area of significant research interest in recent years (31). To investigate the complexity and variability of the TIME, we identified eight immune cell clusters using canonical markers, which included B cells, CD4^+^ T helper cells, CD4^+^ Treg cells, cytotoxic CD8^+^ T cells, NK cells, neutrophils, and M1 and M2 macrophages (**Figure 2A**). As shown in **Figure 2B**, marker genes were identified in different cell clusters using the combined method of SingleR and canonical markers. Specifically, CD8A and CD8B were enriched in cytotoxic CD8^+^ T cells, who mainly exerted antitumor immune killing. Other helper immune cells also play an important role in eliminating tumor cells, including B cells (CD79A), CD4^+^ T helper cells (IL7R and CCR7), NK cells (GNLY and KLRD1), M1 macrophages (CD86), and neutrophils (S100A8). In contrast, tumor cells in the TIME also recruited and induced immune cells to transform into a tumor-promoting phenotype. In our study, the classical immunosuppressive cells consisted of CD4^+^ Treg cells (FOXP3 and CTLA4), and M2 macrophages (CD163 and VSIG4). To effectively explore the heterogeneity of TIME, we further estimated the proportion ratio of each immune cell subpopulation in every patient (**Figure 2C**). The results showed that there was obvious heterogeneity in the immune cell composition among different patients with TNBC. In addition, differentiation trajectory of immune cells was constructed by pseudotime that is a measure of how much progress an individual cell has made during cell differentiation (**Figure 2D**). Based on this differentiation trajectory, it illustrated that myeloid and lymphoid immune cells were differentiated separately, and the differentiation trajectory of immune cells was variable. Collectively, all these results demonstrated the complexity and heterogeneity of the TIME.

### Significance of Macrophages in the Tumor Immune Microenvironment

**Figure 3:**
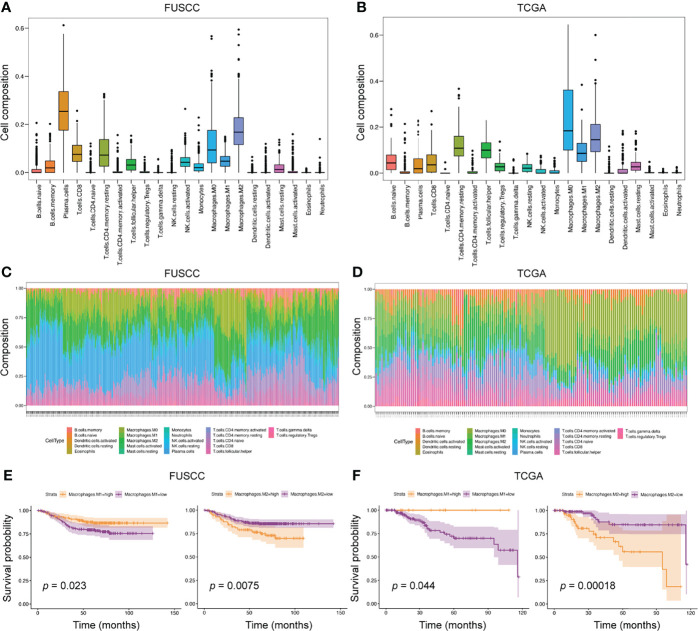
The predominant role of macrophages in TIME from bulk-RNA sequencing analysis. **(A, B)** We applied the ‘CIBERSORT’ package to evaluate the proportion of 22 immune cells in the entire immune microenvironment from 360 FUSCC and 158 TCGA TNBC patients. **(C, D)** In the bulk-RNA sequencing level, the specific proportion of each patients’ immune cell types was calculated to check the heterogeneity of TIME. E-F, Survival analysis was used to calculate the effect of high expression of M1 or M2 macrophages on the prognosis of patients. Cohorts, FUSCC **(E)** and TCGA **(F)**.

Due to the limited sample size of the scRNA-seq analysis only representing a small number of patients, we applied bulk-RNA sequencing from FUSCC and TCGA TNBC cohorts to further dissect the TIME. In this scenario, the ‘CIBERSORT’ package was used to assess the infiltration of various immune cells from 360 FUSCC and 158 TCGA patients with TNBC(**Figures 3A, B**). Consistent with the scRNA-seq results, macrophages accounted for a very high proportion of all 22 immune cells. This result once again emphasized the essential role of macrophages in the TIME. Through calculating the proportion of each patient’s immune cells, we found that the heterogeneity between tumors was widespread in the TIME (**Figures 3C, D**).

To further explore the impact of different immune cell infiltrations on the prognosis of patients, we divided patients into high and low infiltration groups of 22 immune cell types according to the immune cell infiltration score estimated by the ‘CIBERSORT’ package. Then, survival analysis between the two groups was performed. In the FUSCC cohort, five immune cell subtypes were associated with patients’ prognosis, including resting CD4^+^ memory T cells, resting dendritic cells, and M0, M1, and M2 macrophages. However, the TCGA cohort identified seven immune cell subpopulations related to prognostic outcomes, such as activated CD4+ memory T cells, resting NK cells, monocytes, neutrophils, and M0, M1, and M2 macrophages (**Figures 3E, F** and **Supplementary Figures S2A, B**). When comparing the results of the two cohorts, we found that higher M1 macrophages infiltration was associated with better prognosis (*p* = 0.023 and 0.044, respectively), whereas higher M2 macrophages infiltration exhibited shortened survival (*p* = 0.0075 and 0.00018, respectively). Collectively, macrophage subpopulations accounted for a high proportion of TIME, and were related to patients’ prognosis.

### Mutational Landscape Between High and Low Macrophage Infiltration

Generally speaking, macrophages can be induced into M1 anti-tumor or M2 pro-tumor phenotypes. Meanwhile, macrophages in the TIME are not completely dichotomous but follow a dynamic evolution process. As such, we focused on the M1 macrophages, which cause an inflammatory response and present tumor cells to cytotoxic CD8^+^ T cells, eliciting antitumor immunity.

**Figure 4:**
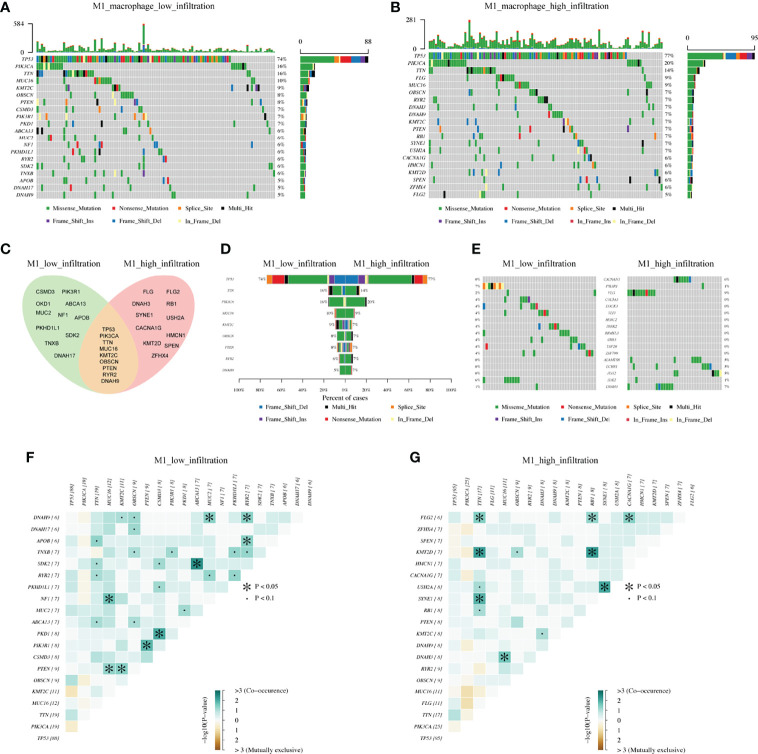
The differentiation of mutational landscape between low and high M1 macrophage infiltration groups from FUSCC cohort. **(A, B)** Top20 mutational genes in M1 macrophage low and high infiltration population. **(C, D)** The overlap of mutations in two groups. **(E)** Different mutations between two groups. **(F, G)** Mutually exclusive or co-occurring mutation in two groups were explored separately.

On this background, we analyzed the mutational landscape between M1 high and low macrophage infiltration groups. The top 20 mutational genes were first identified (**Figures 4A, B**), among which the most prominent variants were TP53 (found in 74% or 77% of tumors), followed by PIK3CA (16% or 20%), and TTN (16% or 14%). When comparing the top 20 mutational genes from the two groups, we found that only nine genes were mutated in common, which included TP53, PIK3CA, TTN, MUC16, KMT2C, OBSCN, PTEN, RYR2, and DNAH9 (**Figures 4C, D**). As shown in **Figure 4, E** we also pointed out differentially mutational genes. In addition, mutually exclusive or co-occurring mutation of top 20 genes were detected (**Figures 4F, G**). The results indicated that PTEN and MUC16 were obviously co-mutated in the M1 low infiltration cohort, but not in the M1 high infiltration cohort. Also, we analyzed the mutational landscape of TCGA cohort (**Supplementary Figure S3**) and M2 infiltration groups (data not shown). Together, these data demonstrated that in TNBC, different mutational landscapes existed between the M1 high and low infiltration patient cohorts.

### Key Marker Genes Associated With M1 Macrophages Infiltration

The above results confirmed that it is possible that the discrepancy in the mutational pattern of tumor cells leads to genetic variation, ultimately shaping the inconsistent macrophage infiltration microenvironment. So which genes are involved in this process?

**Figure 5:**
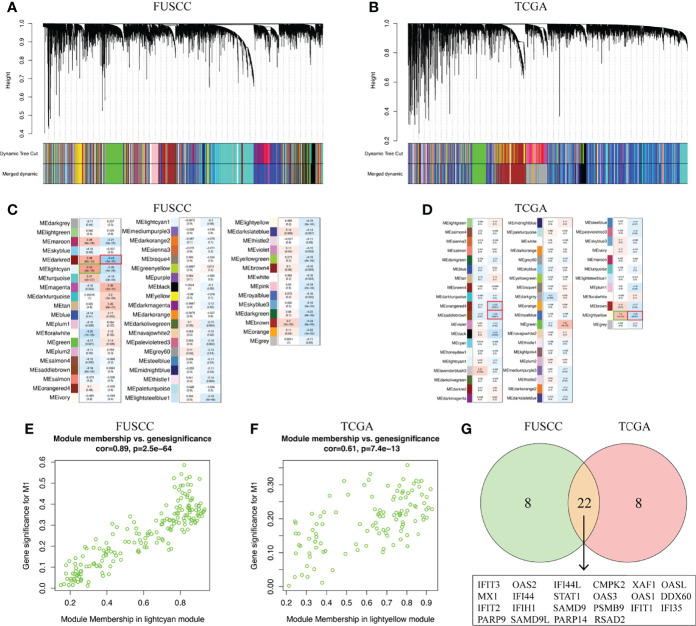
Key marker genes associated with M1 macrophages infiltration was explored. **(A, B)** WGCNA was used to construct a scale-free network with a power of 5 in FUSCC and TCGA cohorts. **(C, D)** The correlation between 54 and 52 gene modules, respectively, and M1 macrophage infiltration were calculated. **(E, F)** Module membership in lightcyan and lighteyellow from FUSCC and TCGA were confirmed to be highly correlated with M1 macrophage infiltration. **(G)** Genes that overlap in lightcyan module from FUSCC and lightyellow module from TCGA.

To identify the key marker genes associated with M1 or M2 macrophages infiltration, WGCNA was used to construct a scale-free network (**Figures 5A, B**). A total of 54 and 52 gene modules were identified from the FUSCC and TCGA TNBC cohorts, among which lightcyan module was associated with higher M1 macrophages infiltration in FUSCC (*r* = 0.44, *p* = 4e^-18^, **Figure 5C**), and lightyellow in TCGA (*r* = 0.3, *p* = 1e^-4^, **Figure 5D**). We further calculated the correlation between module membership and gene significance for M1 macrophages. Both of two modules showed a high correlation with M1 macrophages (cor = 0.89, *p* = 2.5e^-64^; cor = 0.61, *p* = 7.4e^-13^; **Figures 5E, F**). Among these two modules, intramodule connectivity of each genes were estimated, and the top 30 hub genes with the highest degrees were identified. Interestingly, when taking the intersection of top30 hub genes from two modules, we surprisingly found 22 genes were coincident, including IFIT3, IFI35, OAS2, PSMB9, SAMD9L, and so on (**Figure 5G**). Besides we did not find any consistent gene sets in the analysis of the M2 infiltrated groups (**Supplementary Table S2**). Therefore, these 22 genes were likely to be involved in the polarization of M1 macrophages in the TIME.

### Three Typical Genes Associated With M1 Infiltration Were Associated With Better Prognosis

**Figure 6:**
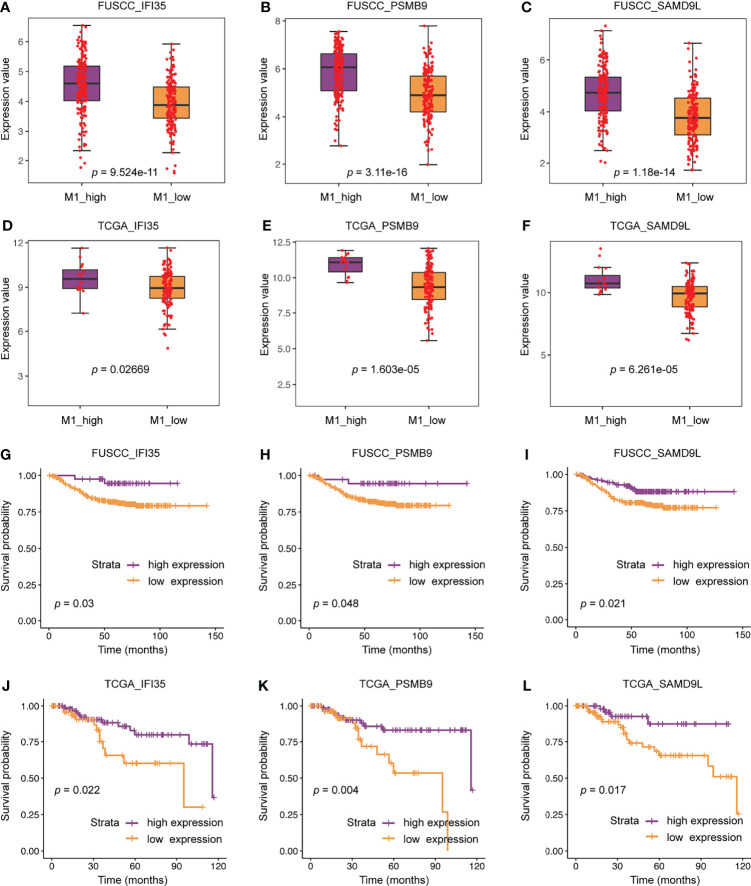
The clinical significance of three representative genes including IFI35, PSMB9, and SAMD9L. **(A–F)** Expression differences of three representative genes between M1 macrophage high and low infiltration groups. **(G–L)** The relationship between three representative genes and patients’ survival.

We further compared the difference in expression of 22 genes between the M1 high and low macrophage infiltration groups. All these genes were higher expression in M1 high macrophage infiltration group from the FUSCC and TCGA datasets (IFI35: *p* = 9.524e^-11^ or 0.02669; PSMB9: *p* = 3.11e^-16^ or 1.603e^-5^; SAMD9L: *p* = 1.18e^-14^ or 6.261e^-5^; **Figures 6A–F**, data not all shown). To assess the relevance of these genes to the patient’s prognosis, survival analysis were used. The results showed that only three representative genes, including IFI35, PSMB9, and SAMD9L, had reached a consistent conclusion in the FUSCC and TCGA cohorts (**Figures 6G–L**). Interestingly, higher expression of IFI35, PSMB9, and SAMD9L was associated with better prognosis (IFI35: *p =* 0.03 or 0.022; PSMB9: *p =* 0.048 or 0.004; SAMD9L: *p =* 0.021 or 0.017), which was consistent with the previous conclusion that patients with higher M1 macrophages had a prolonged survival.

### Exploring the Relationship Between IFI35, PSMB9, and SAMD9L and M1 Macrophages

**Figure 7:**
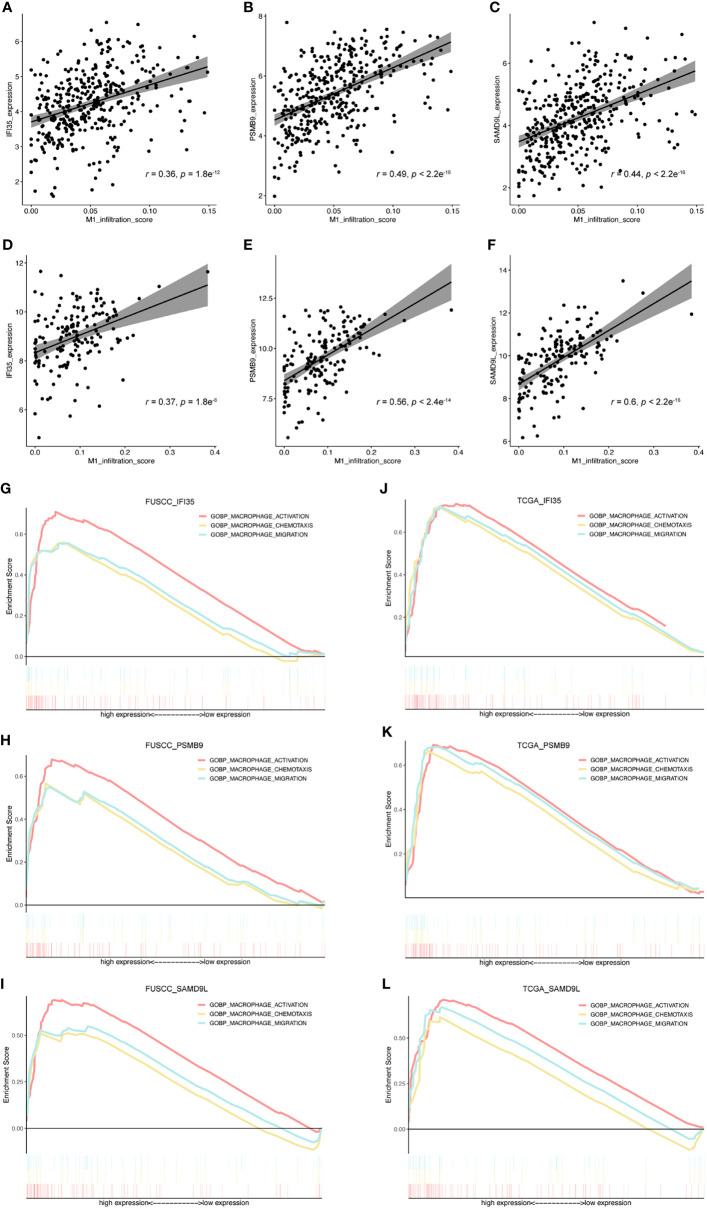
Exploring the relationship between IFI35, PSMB9, and SAMD9L and M1 macrophages. **(A–F)**, The correlation between these 3 genes and M1 macrophage infiltration score. Cohorts, FUSCC **(A–C)** and TCGA **(D, E)**. **(G–L)**, 3 classical macrophage activation, chemotaxis, and migration gene sets were used to explore their relationship with gene expression of IFI35, PSMB9, and SAMD9L.

To further evaluate the connection between IFI35, PSMB9, and SAMD9L and the polarization of macrophages toward the M1 phenotype, we also calculated the correlation between these three genes and M1 macrophage infiltration score. As a result, all the genes were positively correlated with M1 macrophage infiltration (**Figures 7A–F**), in which the correlation coefficient of IFI35 was 0.36 and 0.37 (*p* = 1.8e^-12^ and 1.8e^-6^, respectively), PSMB9 0.49 and 0.56 (*p* = 2.2e^-16^ and 2.4e^-14^, respectively), and SAMD9L 0.44 and 0.6 (*p* = 2.2e^-16^ and 2.2e^-16^, respectively). Moreover, we extracted three classical macrophage activation, chemotaxis and migration gene sets from the GSEA datasets and analyzed the relationship between them and IFI35, PSMB9, and SAMD9L expression (**Figures 7G–L**). Compared with low IFI35 expression, higher IFI35 expression was positively associated with macrophages activation (FUSCC: NES = 2.26, *p*-val <0.001, *q*-val <0.001; TCGA: NES = 2.01, *p*-val <0.001, *q*-val <0.001), chemotaxis (FUSCC: NES = 1.9, *p*-val <0.001, *q*-val <0.001; TCGA: NES = 1.94, *p*-val <0.001, *q*-val <0.001), and migration (FUSCC: NES = 1.98, *p*-val <0.001, *q*-val <0.001; TCGA: NES = 1.89, *p*-val <0.001, *q*-val <0.001). In addition, PSMB9 and SAMD9L also led to the same conclusion with more detailed data shown in **Supplementary Table S3**. A previous study used an *in vivo* macrophage activation model to predict macrophage programs, and 49 gene modules were identified (32). Therefore, we further explored whether higher expression of IFI35, PSMB9, and SAMD9L was associated with these modules. The standard of higher NES, the lower *p*-val and *q*-val (NES > 1, *p*-val < 0.05, *q*-val < 0.25) were considered significant. As expected, approximately 50% of the gene sets were enriched in high expression of these three genes (FUSCC, IFI35: 23/49; PSMB9: 20/49; SAMD9L: 25/49; TCGA, IFI35: 31/49, PSMB9: 34/49, SAMD9L: 21/49; **Supplementary Tables S4A, B**). Taken together, we revealed that three genes that were closely associated with macrophage polarization toward the M1 subtype.

### M1 Polarization of Macrophages Upregulated IFI35 Expression

**Figure 8:**
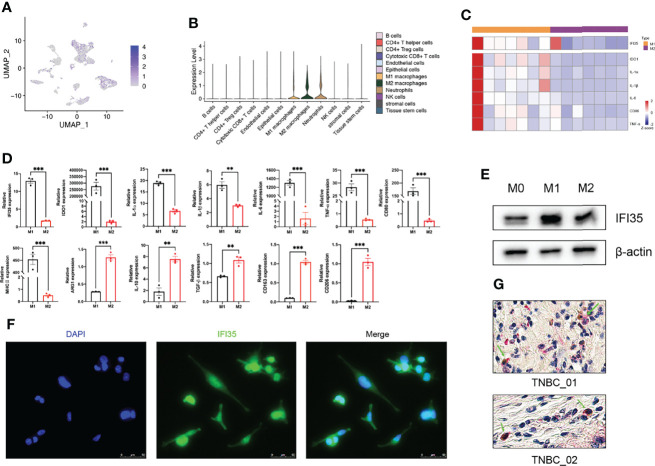
M1 polarization of macrophages upregulated IFI35 expression. **(A, B)**, UMAP and violin plots of IFI35 expression level in each cluster. **(C)**, Heatmap showing differential expression of M1 classical markers and IFI35. **(D)**. qRT-PCR analysis of typical M1 and M2 polarization markers and IFI35 expression in macrophages with different stimuli as indicated. **(E)**, Levels of IFI35 in M0, M1 and M2 macrophages. **(F)**, Representative immunofluorescence images of M1 macrophages stained with DAPI (DNA) and anti-IFI35 antibody. **(G)**. Representative double-labeling IHC images (200×) of TNBC sections stained with CD86 (Brown) and IFI35 (Red). Experiments were performed with at least three biological replicates, and data shown are representative of at least three independent experiments. Data are presented as mean ± SEM. **p < 0.01; ***p < 0.001.

To confirm the results of our bioinformatics analysis, we selected one of the three marker genes for further verification. As we all know, tumor tissue is composed of tumor cells, immune cells, and stromal cells. Therefore, we explored the types of cells in which IFI35 was expressed based on scRNA-seq data. The results indicated that IFI35 was mainly dominated by myeloid-derived cells, including neutrophils, and M1 and M2 macrophages (**Figures 8A, B**). In transcriptome analysis of human M0, M1, and M2 macrophages, M1 macrophages showed higher IFI35 expression than M2 macrophages (**Figure 8C**) (33). In response to different stimuli, M0 macrophages were successfully induced into M1 (LPS and IFN-γ) and M2 (IL-4 and IL-13) macrophages based on a series of M1 typical markers, including IDO1, IL-1α, IL-1β, IL-6, TNF-α, CD80, and MHC II, as well as M2 ones such as ARG1, IL-10, CD163 and CD206 (**Figure 8D**). Obviously, IFI35 was upregulated at both the RNA and protein levels (*p <*0.001, **Figures 8D, E**). Meanwhile, we also demonstrated that IFI35 was expressed in the cytoplasm and nucleus of M1 macrophages, which showed cellular elongation in cell morphology (**Figure 8F**). This conclusion was consistent with the previous study that IFI35 can be transported from the cytoplasm to the nucleus in the activated state (13). Histologically, we found that M1 macrophages with high CD86 expression showed IFI35 expression, which directly reflected the close connection between IFI35 and M1-subtype macrophages (**Figure 8G**).

## Discussion

With the approval of immune checkpoint inhibitors (ICBs) represented by PD-1/PD-L1, anti-tumor therapy has entered a new era of immunotherapy (34). Macrophages, the most abundant immune cell subgroup in the TIME, have received extensive attention. Mounting evidence has revealed that TAMs in TIME-shaped pro-tumor immune responses and were associated with worse clinical outcome in breast cancer, melanoma, lung cancer, and so on (35). Therefore, three methods of TAMs targeting emerged as required so far. Due to the dependence of TAMs on CSF1/CSF1R signaling, targeting CSF1R has become an effective way to deplete macrophages and improve patients’ outcomes (36, 37). In addition, the CCL2-CCR2 axis plays an essential role in the recruitment of classical monocytes to tumor sites. Thus, inhibition of TAMs recruitment by targeting CCL2 or CCR2 has successfully reduced tumor burden in melanoma, breast cancer, and so on. However, the depletion of macrophages also brings a series of side effects that damage tissue homeostasis. Therefore, reprogramming of TAMs has become a more promising treatment strategy. Specifically, it mainly include toll‐like receptor agonist, CD40 agonists, PI3Kγ inhibitors, inhibition of microRNA activity, anti‐CD47 antibodies, and anti‐MARCO antibody therapy (38–40). Certain efficacies have been documented. Thus, the exploration for new molecular targets regulating TAMs repolarization is an attractive direction for cancer immunotherapy.

This study not only revealed the intra- and inter-tumor heterogeneity in TNBC, more importantly, demonstrated the complexity of the TIME and explored an abundance of macrophages using the scRNA-seq data of five patients, and two bulk-RNA sequencing datasets consisting of 158 patients from TCGA and 360 patients from our cohort. From multiple perspectives, it has been fully demonstrated that macrophages were the most abundant immune cell subset in the TIME, and the infiltration of M1 macrophages were associated with a better prognosis. This suggested that awakening the function of M1 macrophages from the overall macrophage population in TNBC would bring prolonged survival. Additionally, three representative genes including IFI35, PSMB9, and SAMD9L showed positive correlation with M1 macrophages. Thus, we speculated that these genes may participate in macrophage polarization toward the M1 subtype.

IFI35 has been widely studied in inflammation-related diseases (IRDs) and antiviral immunity since its discovery in 1993 (12). In IRDs, a large number of studies have pointed out that IFI35 was a marker of inflammation. Specifically, it promoted the progression of nephritis by activating the JAK-STAT1 signaling pathway, and served as a DAMP to activate NF-κB pathway, leading to the exacerbation of neuroinflammation (18). However, it seemed to be a double-edged sword. On the one hand, IFI35 can bind with Nmi or BTas after virus infection, thereby activating type I interferon antiviral response (14). On the other hand, it also negatively regulate RIG-I-mediated antiviral signaling *via* K48-linked ubiquitination (15). Then, what role does IFI35 play in the local inflammatory microenvironment after tumor formation, pro-tumor or anti-tumor role, is worthy of our further study.

To date, in the field of tumor biology, only a few studies revealed that IFI35 promoted the radiosensitivity of lung adenocarcinoma and colorectal cancer (21, 22). Herein, we explored the positive correlation between IFI35 expression and M1 infiltration in TNBC from bioinformatics analysis. Since IFI35 is an interferon-inducible protein, and interferon can induce M1-subtype polarization of macrophages, we speculated that IFI35 is upregulated with M1 subtype differentiation of macrophages. From two levels of the cell line and tissue sections of TNBC, we confirmed this hypothesis (**Figure 8**). Given the anti-tumor function of M1 macrophages and the better prognosis of patients with high IFI35 expression (**Figures 6G, J**), it suggested that IFI35 plays a pro-inflammatory and anti-tumor role in the TIME. However, the exact role of IFI35 in macrophages requires further validation in knockout mice, which is the focus of our next study.

In summary, this study has uncovered the essential role of macrophages. For the first time, the link between IFI35 and M1 macrophages in TNBC was elucidated, and the anti-tumor role of IFI35 was confirmed. Thus, IFI35 may be a promising novel target that helps to reshape macrophage polarization for antitumor effects.

## Data Availability Statement

Publicly available datasets were analyzed in this study. This data can be found here: the raw data were obtained from publicly available datasets (European Genome-phenome Archive with the study ID EGAS00001005061, TCGA) and our FUSCC datasets have been uploaded to the National Omics Data Encyclopedia (OEP000155).

## Ethics Statement

The studies involving human participants were reviewed and approved by the Ethics Committee of Fudan University Shanghai Cancer Center (050432-4-1805C). The patients/participants provided their written informed consent to participate in this study.

## Author Contributions

WJ and BX designed this project. BX, XS, and QL completed the relevant experiment. BX and XS completed the process of analysis. BX completed the draft. HS and WJ reviewed this manuscript. All authors contributed to the article and approved the submitted version.

## Funding

This work was supported by the grant from National Natural Science Foundation of China (81972727).

## Conflict of Interest

The authors declare that they have no competing interests.

## Publisher’s Note

All claims expressed in this article are solely those of the authors and do not necessarily represent those of their affiliated organizations, or those of the publisher, the editors and the reviewers. Any product that may be evaluated in this article, or claim that may be made by its manufacturer, is not guaranteed or endorsed by the publisher.

## Supplementary Material

Supplementary Figure 1 12 cell clusters were annotated, which included multiple immune cell clusters, epithelial cells, stromal cells, endothelial cells, and tissue stem cells.

Supplementary Figure 2 Survival analysis of immune cell subsets related to patients’ prognosis. Cohorts, FUSCC **(A)** and TCGA **(B)**.

Supplementary Figure 3 The differentiation of mutational landscape between low and high M1 macrophage infiltration groups from TCGA cohort. **(A, B)**, Top20 mutational genes in M1 macrophage low and high infiltration population. **(C, D)**, The overlap of mutations in two groups. **(E)**, Different mutations between two groups. **(F, G)**, Mutually exclusive or co-occurring mutation in two groups were explored separately.
