# Supplementary figures and images for: Mapping the Tumor Microenvironment in TNBC and Deep Exploration for M1 Macrophages-Associated Prognostic Genes
Source: Front Immunol. 2022 Jun 30;13:923481. doi: 10.3389/fimmu.2022.923481 (PMC9279655; doi:10.3389/fimmu.2022.923481)

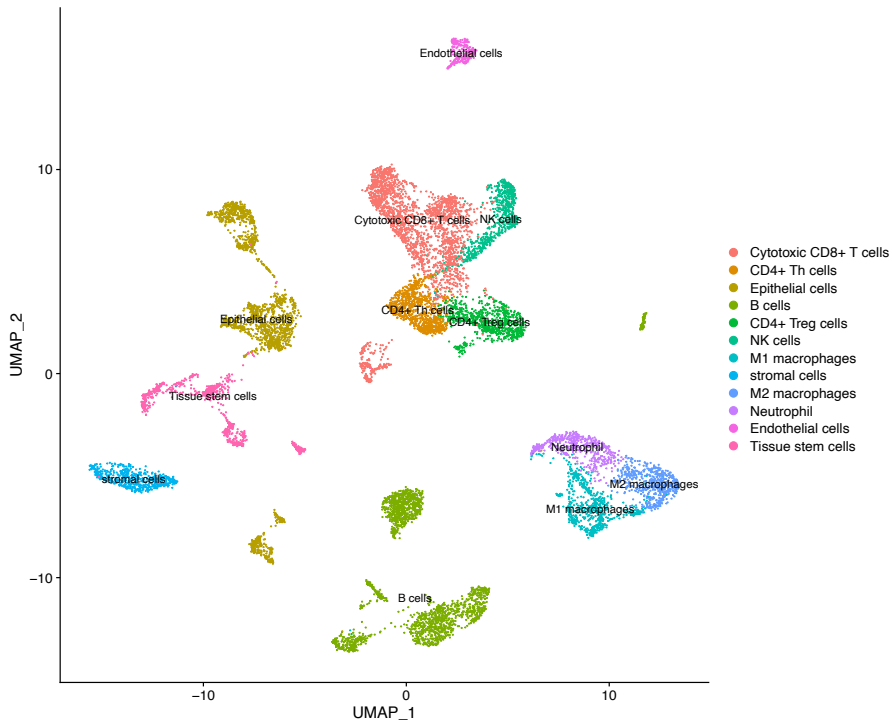

Supplement: Supplementary Figure 1 — 12 cell clusters were annotated, which included multiple immune cell clusters, epithelial cells, stromal cells, endothelial cells, and tissue stem cells. [file DataSheet_1.pdf]

A

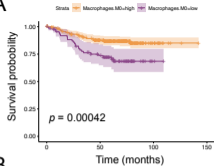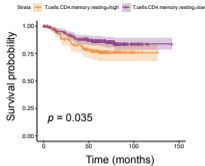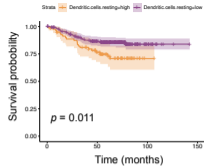

B

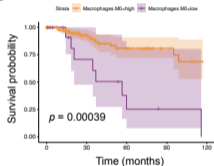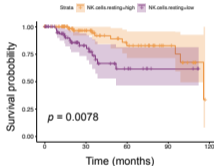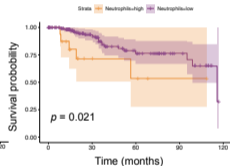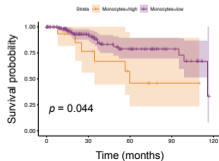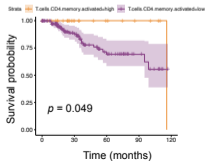

Supplement: Supplementary Figure 2 — Survival analysis of immune cell subsets related to patients’ prognosis. Cohorts, FUSCC (A) and TCGA (B). [file DataSheet_2.pdf]
