# Supplementary material for: Mapping the Tumor Microenvironment in TNBC and Deep Exploration for M1 Macrophages-Associated Prognostic Genes
Source: Front Immunol. 2022 Jun 30;13:923481. doi: 10.3389/fimmu.2022.923481 (PMC9279655; doi:10.3389/fimmu.2022.923481)
Supplement: Supplementary Figure 3 — The differentiation of mutational landscape between low and high M1 macrophage infiltration groups from TCGA cohort. (A, B), Top20 mutational genes in M1 macrophage low and high infiltration population. (C, D), The overlap of mutations in two groups. (E), Different mutations between two groups. (F, G), Mutually exclusive or co-occurring mutation in two groups were explored separately. [file DataSheet_3.pdf]

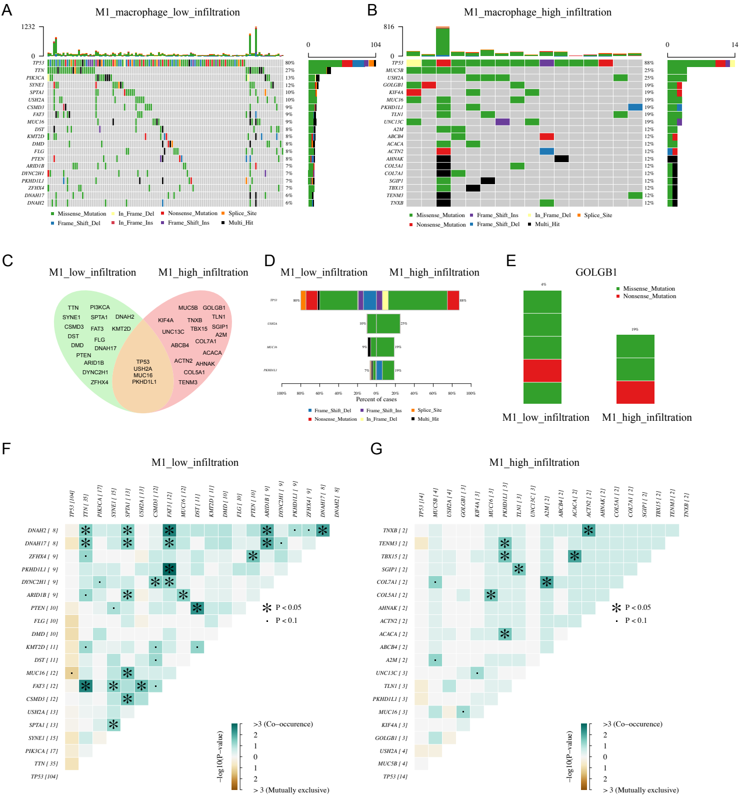

A
M1_macrophage_low_infiltration
1232
0
104
TP53 80%
TTN 27%
PIK3CA 13%
SYNE1 12%
SPTA1 10%
USH2A 10%
CSMD3 9%
FAT3 9%
MUC16 9%
DST 8%
KMT2D 8%
DMD 8%
FLG 8%
PTEN 8%
ARID1B 7%
DYNC2H1 7%
PKHD1L1 7%
ZFHX4 7%
DNAH17 6%
DNAH2 6%
Missense_Mutation
In_Frame_Del
Nonsense_Mutation
Splice_Site
Frame_Shift_Del
In_Frame_Ins
Frame_Shift_Ins
Multi_Hit
B
M1_macrophage_high_infiltration
816
0
14
TP53 88%
MUC5B 25%
USH2A 25%
GOLGB1 19%
KIF4A 19%
MUC16 19%
PKHD1L1 19%
TLN1 19%
UNC13C 19%
A2M 12%
ABCB4 12%
ACACA 12%
ACTN2 12%
AHNAK 12%
COL5A1 12%
COL7A1 12%
SGIP1 12%
TBX15 12%
TENM3 12%
TNXB 12%
Missense_Mutation
Frame_Shift_Ins
In_Frame_Del
Splice_Site
Nonsense_Mutation
Frame_Shift_Del
Multi_Hit
C
M1_low_infiltration
M1_high_infiltration
TTN PI3KCA SYNE1 SPTA1 DNAH2 CSMD3 FAT3 KMT2D DST FLG DMD DNAH17 PTEN ARID1B DYNC2H1 ZFHX4
TP53 USH2A MUC16 PKHD1L1
MUC5B GOLGB1 KIF4A TNXB TLN1 UNC13C TBX15 SGIP1 A2M ABCB4 COL7A1 ACACA ACTN2 AHNAK COL5A1 TENM3
D
M1_low_infiltration
M1_high_infiltration
80% 88%
10% 25%
9% 19%
7% 19%
100% 80% 60% 40% 20% 0% 20% 40% 60% 80% 100%
Percent of cases
Frame_Shift_Del
Frame_Shift_Ins
Splice_Site
Nonsense_Mutation
In_Frame_Del
Multi_Hit
E
GOLGB1
Missense_Mutation
Nonsense_Mutation
4%
19%
M1_low_infiltration
M1_high_infiltration
F
M1_low_infiltration
TP53 [104]
TTN [35]
PIK3CA [17]
SYNE1 [15]
SPTA1 [13]
USH2A [13]
CSMD3 [12]
FAT3 [12]
MUC16 [12]
DST [11]
KMT2D [11]
DMD [10]
FLG [10]
PTEN [10]
ARID1B [9]
DYNC2H1 [9]
PKHD1L1 [9]
ZFHX4 [9]
DNAH17 [8]
DNAH2 [8]
P < 0.05
P < 0.1
>3 (Co−occurence)
2
1
0
1
2
> 3 (Mutually exclusive)
−log10(P−value)
G
M1_high_infiltration
TP53 [14]
MUC5B [4]
USH2A [4]
GOLGB1 [3]
KIF4A [3]
MUC16 [3]
PKHD1L1 [3]
TLN1 [3]
UNC13C [3]
A2M [2]
ABCB4 [2]
ACACA [2]
ACTN2 [2]
AHNAK [2]
COL5A1 [2]
COL7A1 [2]
SGIP1 [2]
TBX15 [2]
TENM3 [2]
TNXB [2]
P < 0.05
P < 0.1
>3 (Co−occurence)
2
1
0
1
2
> 3 (Mutually exclusive)
−log10(P−value)
